# A Shoot Fe Signaling Pathway Requiring the OPT3 Transporter Controls GSNO Reductase and Ethylene in *Arabidopsis thaliana* Roots

**DOI:** 10.3389/fpls.2018.01325

**Published:** 2018-09-11

**Authors:** María J. García, Francisco J. Corpas, Carlos Lucena, Esteban Alcántara, Rafael Pérez-Vicente, Ángel M. Zamarreño, Eva Bacaicoa, José M. García-Mina, Petra Bauer, Francisco J. Romera

**Affiliations:** ^1^Department of Botany, Ecology and Plant Physiology, Campus de Excelencia Internacional Agroalimentario, Universidad de Córdoba, Córdoba, Spain; ^2^Department of Biochemistry, Cell and Molecular Biology of Plants, Estación Experimental del Zaidín, Spanish National Research Council, Granada, Spain; ^3^Department of Agronomy, Campus de Excelencia Internacional Agroalimentario, Universidad de Córdoba, Córdoba, Spain; ^4^Department of Environmental Biology, Faculty of Sciences, University of Navarra, Pamplona, Spain; ^5^Institute of Botany, University of Düsseldorf, Düsseldorf, Germany

**Keywords:** ethylene, glutathione (GSH), iron, long distance iron signal (LODIS), nitric oxide (NO), phloem, *S*-nitrosoglutathione (GSNO), *S*-nitrosoglutathione reductase (GSNOR)

## Abstract

Ethylene, nitric oxide (NO) and glutathione (GSH) increase in Fe-deficient roots of Strategy I species where they participate in the up-regulation of Fe acquisition genes. However, *S*-nitrosoglutathione (GSNO), derived from NO and GSH, decreases in Fe-deficient roots. GSNO content is regulated by the GSNO-degrading enzyme *S*-nitrosoglutathione reductase (GSNOR). On the other hand, there are several results showing that the regulation of Fe acquisition genes does not solely depend on hormones and signaling molecules (such as ethylene or NO), which would act as activators, but also on the internal Fe content of plants, which would act as a repressor. Moreover, different results suggest that total Fe in roots is not the repressor of Fe acquisition genes, but rather the repressor is a Fe signal that moves from shoots to roots through the phloem [hereafter named LOng Distance Iron Signal (LODIS)]. To look further in the possible interactions between LODIS, ethylene and GSNOR, we compared *Arabidopsis* WT Columbia and LODIS-deficient mutant *opt3-2* plants subjected to different Fe treatments that alter LODIS content. The *opt3-2* mutant is impaired in the loading of shoot Fe into the phloem and presents constitutive expression of Fe acquisition genes. In roots of both Columbia and *opt3-2* plants we determined 1-aminocyclopropane-1-carboxylic acid (ACC, ethylene precursor), expression of ethylene synthesis and signaling genes, and GSNOR expression and activity. The results obtained showed that both ‘ethylene’ (ACC and the expression of ethylene synthesis and signaling genes) and ‘GSNOR’ (expression and activity) increased in Fe-deficient WT Columbia roots. Additionally, Fe-sufficient *opt3-2* roots had higher ‘ethylene’ and ‘GSNOR’ than Fe-sufficient WT Columbia roots. The increase of both ‘ethylene’ and ‘GSNOR’ was not related to the total root Fe content but to the absence of a Fe shoot signal (LODIS), and was associated with the up-regulation of Fe acquisition genes. The possible relationship between GSNOR(GSNO) and ethylene is discussed.

## Introduction

Iron (Fe) is abundant in most soils, mainly as Fe^3+^, although its availability to plants is low, especially in calcareous soils (Römheld and Marschner, 1986). Based on the mechanisms used by plant roots to facilitate mobilization and uptake of Fe, plants are classified into Strategy I species and Strategy II species (Römheld and Marschner, 1986; Ivanov et al., 2012; Kobayashi and Nishizawa, 2012). Dicots, such as *Arabidopsis* and pea (*Pisum sativum*), are Strategy I species and reduce Fe^3+^ to Fe^2+^, by means of a ferric reductase (encoded by *FRO2* in *Arabidopsis*) at the root surface, prior to its subsequent uptake through a Fe^2+^ transporter (encoded by *IRT1* in *Arabidopsis*; Ivanov et al., 2012; Kobayashi and Nishizawa, 2012). When grown under Fe deficiency, Strategy I species develop several physiological and morphological responses, mainly in roots, which favor Fe acquisition and are generally known as Fe deficiency responses. Among the physiological responses are the up-regulation of the ferric reductase and the Fe^2+^ transporter genes, as well as many other Fe-related genes (Ivanov et al., 2012; Kobayashi and Nishizawa, 2012; Brumbarova et al., 2015; Lucena et al., 2015). In the last years, several transcription factors (TFs) that participate in the activation of these genes have been found (Ivanov et al., 2012; Kobayashi and Nishizawa, 2012; Brumbarova et al., 2015; Zhang et al., 2015; Li et al., 2016; Liang et al., 2017). In *Arabidopsis*, the master regulator of most of the Fe-related genes is FIT (bHLH29), homolog of the tomato FER gene (Bauer et al., 2007 and references therein). The FIT regulatory network comprises other bHLH TFs of the Ib subgroup, such as bHLH38, bHLH39, bHLH100, and bHLH101. All of them have redundant functions and can interact with FIT to form heterodimers that activate the expression of the Fe acquisition genes *FRO2* and *IRT1* (Yuan et al., 2008; Wang et al., 2013; Brumbarova et al., 2015). FIT is induced in roots in response to Fe deficiency while the other Ib bHLH genes cited above are induced in both roots and leaves in response to Fe deficiency (Brumbarova et al., 2015 and references therein). Lately, it has been found that, under Fe-deficiency conditions, IVc subgroup bHLH TFs, like bHLH105 and bHLH115, activate the expression of FIT/bHLH38/39/100/101 (Zhang et al., 2015; Li et al., 2016; Liang et al., 2017).

The regulation of the above TFs and genes is not totally understood, but several works support a role for hormones and signaling molecules in the activation of FIT (and other Ib bHLH TFs) and, consequently, in the up-regulation of the ferric reductase and the Fe^2+^ transporter genes. Among them are auxin, ethylene, salicylic acid, nitric oxide (NO), sucrose, and glutathione (GSH). All of them increase in Fe-deficient roots although their precise roles and interactions are not totally known (Zaharieva and Abadía, 2003; Zaharieva et al., 2004; Lucena et al., 2006, 2015; Graziano and Lamattina, 2007; Waters et al., 2007; Bacaicoa et al., 2009, 2011; Chen et al., 2010; García et al., 2010, 2011; Lingam et al., 2011; Meiser et al., 2011; Romera et al., 2011, 2017; Koen et al., 2012; Yang et al., 2014; Shanmugam et al., 2015; Lin et al., 2016; Shen et al., 2016; Li and Lan, 2017; Kailasam et al., 2018). There are also some hormones, such as cytokinins and jasmonates, that have been involved in the suppression of Fe deficiency responses (Séguéla et al., 2008; Maurer et al., 2011). Besides the activation of Fe-related genes, auxin, ethylene, and NO have also been involved in the regulation of morphological responses to Fe deficiency, such as the development of subapical root hairs (Romera et al., 2011, 2017; Lucena et al., 2015; Li and Lan, 2017). Additionally, ethylene is involved in restricting the suberization of the endodermis under Fe deficiency (Barberon et al., 2016), and NO and GSH are involved in improving Fe availability inside plants (Graziano et al., 2002; Ramírez et al., 2013).

In a previous work, García et al. (2011) showed that ethylene can induce NO accumulation in the subapical region of the roots, where most Fe responses are located. On the other hand, they found that NO can upregulate many ethylene synthesis and signaling genes in roots. This mutual and positive influence between ethylene and NO has also been described in the development of root hairs under Mg deficiency (Liu et al., 2017) and in other physiological processes (García et al., 2011 and references therein; Lin et al., 2013). Both ethylene and NO greatly activate the expression of Fe acquisition genes in plants grown with low levels of Fe (or without Fe), but have much less effect in plants grown with high levels of Fe (Lucena et al., 2006; Graziano and Lamattina, 2007; Chen et al., 2010; García et al., 2011). Similar results have also been found when other activators of Fe acquisition genes, like auxin (Chen et al., 2010) or sucrose (Lin et al., 2016), have been applied to plants grown with different Fe levels. All these results suggest that the up-regulation of Fe acquisition genes does not solely depend on hormones and signaling molecules (such as ethylene, auxin, sucrose, or NO), that would act as activators, but also on the internal Fe content of plants, that would act as a repressor (Lucena et al., 2006; García et al., 2011, 2013; Romera et al., 2011). However, different results suggest that total Fe in roots is not the repressor of Fe acquisition genes. As examples, *Arabidopsis opt3, frd3, nas4x-1* and *bts3* mutants, *Arabidopsis AtHSCB* overexpressing lines, tomato *chln* mutant, and pea *dgl* and *brz* mutants, all of them present constitutive activation of Fe acquisition genes when grown under Fe sufficiency despite the high accumulation of Fe inside their roots (García et al., 2013 and references therein; Romera et al., 2015; Leaden et al., 2016; Hindt et al., 2017; Khan et al., 2018). Several of the above genotypes are affected, either directly or indirectly, in the transport of Fe through the phloem, which suggests that phloem Fe could be a key factor in the repression of Fe acquisition genes (García et al., 2013; Khan et al., 2018). In any case, there is no doubt that shoots play a very important role in the regulation of Fe acquisition genes in roots (García et al., 2013 and references therein; Mendoza-Cózatl et al., 2014; Zhai et al., 2014; Gayomba et al., 2015). In accordance with this, Khan et al. (2018) have recently demonstrated that the leaf vasculature respond to Fe deficiency considerably faster than roots, and Kumar et al. (2017) have found, by using reciprocal shoot/root grafts between *Arabidopsis ysl1ysl3* double mutant and WT plants, that the ability to express the Fe deficiency responses depends on the genotype of the shoot.

OPT3 (OligoPeptide Transporter3) is the main transporter implicated in the loading of shoot Fe into the phloem (Mendoza-Cózatl et al., 2014; Zhai et al., 2014; Kumar et al., 2017; Khan et al., 2018). In supporting this view, it should be noted that the foliar application of Fe did not inhibit the expression of Fe acquisition genes in Fe-deficient *Arabidopsis opt3-2* roots while it did in Fe-deficient *Arabidopsis* WT roots (García et al., 2013). Furthermore, phloem sap measurements in *opt3* mutants show a 50% reduction in Fe content (Khan et al., 2018). *OPT3* is expressed in the plasma membrane of phloem cells, mainly in shoots, and its shoot-specific expression is sufficient to complement the Fe-deficiency response in *opt3-2* roots (Mendoza-Cózatl et al., 2014; Zhai et al., 2014; Gayomba et al., 2015). These results suggest that OPT3 plays an important role in the iron-signaling network between leaves and roots (García et al., 2013; Mendoza-Cózatl et al., 2014; Zhai et al., 2014; Gayomba et al., 2015). The precise substrate of the OPT3 transporter is not yet clear. Zhai et al. (2014) found that OPT3 can transport Fe^2+^ ions when expressed in *Xenopus* oocytes. However, while Wintz et al. (2003) found that OPT3 was able to rescue the *fet3fet4* strain of yeast, impaired in Fe uptake, Mendoza-Cózatl et al. (2014) found that not. Even if OPT3 transports Fe ions, these ions should be chelated in the phloem sap due to the poor solubility of Fe at the alkaline pH of this fluid (Gutiérrez-Carbonell et al., 2015). Although OPT3 does not mediate GSH transport in *S. cerevisiae* (Zhai et al., 2014), other OPT transporters do (Lubkowitz, 2011 and references therein; Zhang et al., 2016 and references therein). Consequently, some proposed chelating agents of Fe are GSH-derived compounds, like *S*-nitrosoglutathione (GSNO) or nitrosyl-iron complexes (NICs, formed from the interaction of Fe, NO and thiols); also peptides and proteins (Krüger et al., 2002; Ramírez et al., 2011; Rahmanto et al., 2012; Darbani et al., 2013; García et al., 2013; Buet and Simontacchi, 2015). Based on these findings, we suggest the name LODIS (LOng Distance Iron Signal) for the shoot Fe signal related to OPT3 and moving through the phloem, which causes the repression of Fe acquisition genes in roots. Although the LODIS nature is still unknown, it is possible to investigate its consequences by comparing the LODIS-deficient mutant *opt3-2* and the WT cultivar Columbia, and by comparing plants treated or not with foliar application of Fe.

Another important question, which remains unsolved, is how the Fe status is perceived by the roots. At this point, it has been proposed that Fe (probably, LODIS or a LODIS-derived signal) could be sensed by the BRUTUS (BTS) protein in Strategy I plants (Kobayashi and Nishizawa, 2014). BTS is a homolog of HRZs, known Fe sensors in rice (Kobayashi and Nishizawa, 2014). The current data suggests that BTS acts as a negative regulator of Fe responses (Zhang et al., 2015; Hindt et al., 2017). This would explain why Fe-sufficient *bts-2* and *bts-3* roots present higher expression of Fe acquisition genes than Fe-sufficient WT roots (Zhang et al., 2015; Hindt et al., 2017).

To integrate both positive and negative signals in the regulation of Fe acquisition genes in roots, Lucena et al. (2006) proposed a model that implicated both LODIS and ethylene in such a regulation. Subsequently, this model has been extended to other positive signals besides ethylene, like NO and auxin (García et al., 2011; Romera et al., 2011, 2017). According to this model, auxin/ethylene/NO would act as activators of the expression of Fe acquisition genes in roots, while LODIS would act to repress their expression (García et al., 2011; Romera et al., 2011, 2017). This model does not exclude the role of other positive and negative signals (most of them reviewed by García et al., 2015; Romera et al., 2017). The way LODIS represses the expression of Fe acquisition genes is not totally known, but several results suggest that it could negatively affect ethylene action. García et al. (2013) found that ACC up-regulated the expression of Fe acquisition genes when applied to roots of Fe-deficient plants but not when applied simultaneously with foliar Fe. This result suggests that LODIS could block ethylene action, but does not preclude an additional negative effect of LODIS on ethylene production.

One of the objectives of this work was to study the role of LODIS on ethylene synthesis in roots. For this, we determined 1-aminocyclopropane-1-carboxylic acid (ACC) concentration and the expression of the key ethylene synthesis genes *SAM1, ACS*6, and *ACO2* in roots of *Arabidopsis* WT Columbia plants subjected to different Fe treatments that alter LODIS content. It should be noted that ACC content and the expression of the above ethylene synthesis genes increase under Fe deficiency (García et al., 2010; Gutiérrez-Carbonell et al., 2015; Lucena et al., 2015 and references therein; Ye et al., 2015; Romera et al., 2017 and references therein). In a parallel study, similar determinations were done in the *Arabidopsis* LODIS-deficient mutant *opt3-2*, and in other *Arabidopsis* mutants that behave like LODIS-deficient, such as *frd3-3* and *nas4x-1*. All of these mutants have constitutive activation of Fe acquisition genes when grown under Fe sufficient conditions (see section “Materials and Methods”; Rogers and Guerinot, 2002; Klatte et al., 2009; García et al., 2013). A second objective of this work was to study the possible relationship of *S*-nitrosoglutathione reductase (GSNOR) with Fe deficiency and, more specifically, with LODIS. Both GSH and NO increase in roots under Fe deficiency and have been implicated in the activation of Fe responses (Zaharieva and Abadía, 2003; Zaharieva et al., 2004; Graziano and Lamattina, 2007; Bacaicoa et al., 2009; Chen et al., 2010; García et al., 2011; Koen et al., 2012; Shanmugam et al., 2015; Kailasam et al., 2018). However, *S*-nitrosoglutathione (GSNO), the most abundant low-molecular-weight *S*-nitrosothiol in plants (Liu et al., 2018), and which is derived from GSH and NO (Corpas et al., 2013), decreases under Fe deficiency in *Arabidopsis* roots (Shanmugam et al., 2015; Kailasam et al., 2018). Since GSNOR regulates GSNO content by decomposing it to oxidized glutathione (GSSG) and H_3_N (Leterrier et al., 2011; Corpas et al., 2013), we wanted to know whether GSNOR increases in roots under Fe deficiency. For this, we determined GSNOR expression and activity under Fe deficiency and also under different Fe treatments and genetic backgrounds that could influence LODIS levels.

The results show that ethylene (ACC and the expression of ethylene synthesis genes) and GSNOR (expression and activity) increase under conditions that restrict LODIS accumulation in roots, such as Fe deficiency in WT or Fe sufficiency in the LODIS-deficient mutant *opt3-2*, while they decrease under conditions that favor its accumulation, such as Fe sufficiency or foliar application of Fe in WT. The up-regulation of GSNOR under Fe deficiency in the WT suggests that lower GSNO levels in roots could be a prerequisite for the up-regulation of ethylene synthesis and, consequently, for that of Fe acquisition genes.

## Materials and Methods

### Plant Materials, Growth Conditions, and Treatments

To study the role of LODIS on ethylene and GSNOR in roots, wild-type *Arabidopsis* [*Arabidopsis thaliana* (L.) Heynh ecotype Columbia] and pea [*Pisum sativum* L. cv. Sparkle] plants were used. Additionally, we used some mutants that show constitutive up-regulation of Fe acquisition genes when grown under Fe-sufficient conditions and that are, or behave like, LODIS-deficient (García et al., 2013 and references therein). These mutants were *Arabidopsis opt3-2, frd3-3* and *nas4x-1*, and pea *dgl* (Sparkle [*dgl,dgl*]). *OPT3* is expressed mainly in shoots, where it could act as a transporter involved in the loading of Fe^2+^ ions into the phloem (Stacey et al., 2008; Zhai et al., 2014; see **Figure 12**). The *Arabidopsis frd3-3* mutant is impaired in xylem Fe transport (Rogers and Guerinot, 2002; Roschzttardtz et al., 2011; Gayomba et al., 2015) but, as a consequence, it is also defective in the transport of Fe from shoots to roots through the phloem (it behaves like LODIS-deficient), since less Fe gets into leaves to enter the phloem (Lucena et al., 2006; see **Figure 12**). The *Arabidopsis nas4x-1* mutant is defective in the synthesis of NA, which is involved in Fe loading and unloading of the phloem (Klatte et al., 2009; Schuler et al., 2012). Both *frd3-3* and *nas4x-1* mutants show chlorosis when grown under Fe-sufficient conditions (Lucena et al., 2006; Klatte et al., 2009). In relation to the pea *dgl* mutant, the specific gene related to this mutation has not been identified yet. However, several results suggest that the *dgl* mutant phenotype may be related to defects in phloem Fe transport since it behaves like a LODIS-deficient mutant (García et al., 2013; Romera et al., 2015). Although the *dgl* mutant originated from the DGV (Dippes Gelbe Viktoria) cultivar, we used a near isogenic line homozygous for the *dgl* mutation that was introgressed into the Sparkle cultivar and described here as Sparkle [*dgl, dgl*] (Marentes and Grusak, 1998).

*Arabidopsis* and pea plants were grown on aerated nutrient solution in a growth chamber, as previously described (Lucena et al., 2006, 2007). When appropriate, plants were transferred to the different treatments. The treatments imposed were: **+Fe**: nutrient solution with 40 μM Fe-EDDHA, except for pea WT Sparkle, that was 20 μM Fe-EDDHA, and for pea *dgl*, that was 3 μM Fe-EDDHA (this mutant grows adequately with 2–3 μM Fe-EDDHA concentration but presents symptoms of Fe toxicity when grown with higher levels of Fe; Romera et al., 2015); -**Fe**: nutrient solution without Fe during different times (from 6 to 72 h, depending on experiments); -**Fe+foliarFe**: -Fe treatment during 2 or 3 days and FeSO_4_ application to leaves during the last 24 h; -**P**: nutrient solution without P during 2 days; -**S**: nutrient solution without S during 2 days. It should be noted that, in our experimental conditions, the expression of Fe acquisition genes and the ferric reductase activity was weak after 24 h of Fe deficiency and reached their maximum after 48 h of Fe deficiency. FeSO_4_ was dissolved in deionized water (1.8 mM) and Tween 20 was added as surfactant. Leaves were sprayed once until total moistening. After treatments, root ferric reductase activity was determined as described previously (Lucena et al., 2006). Finally, the roots were collected and kept at -80°C for subsequent analysis of ACC and mRNA levels. In some experiments, GSNOR activity, GSNO and GSH were determined in fresh roots.

### ACC Determination

The extraction, purification and quantification of ACC was carried out using the method described by Mora et al. (2012). Briefly, ACC of roots was extracted with 20 μl of d_4_ACC [3 μg/mL in acetonitrile/acetic acid 0.2% (90/10)] and 3 ml of MeOH/H_2_O/HCOOH (15/4/1, v/v/v) at -20°C. Purification was carried out using a Strata C18-E cartridge (Ref 8B-S001-FBJ, Phenomenex, Torrance, CA, United States) preconditioned with 4 ml of methanol and 2 ml of MeOH/H_2_O/HCOOH (15/4/1, v/v/v). Finally, the eluted fraction was centrifuged (10,000 rpm, 8 min) and injected in the LC/MS/MS systems, and ACC was quantified by HPLC linked to a 3200 QTRAP LC/MS/MS system (Applied Biosystems/MDS Sciex, Ontario, Canada), equipped with a turbo ion spray interface.

### GSNOR Activity Determination

GSNOR activity was assayed spectrophotometrically at 25°C by monitoring the oxidation of NADH at 340 nm as described by Sakamoto et al. (2002). The root extracts were incubated in an assay mixture containing 20 mM TRIS-HCl (pH 8.0), 0.2 mM NADH, and 0.5 mM EDTA, and the reaction was started by adding GSNO (Calbiochem) to the mixture at a final concentration of 400 μM. The activity was expressed as nmol NADH consumed min^-1^ mg^-1^ protein (𝜀_340_ = 6.22 mM^-1^ cm^-1^).

### Quantification of GSNO and GSH by Liquid Chromatography Electrospray Ionization Mass Spectrometry (LC–ES/MS)

*Arabidopsis* samples (300 mg) were ground using a mortar and pestle in the presence of 1 ml of 0.1 M HCl. Homogenates were centrifuged at 15,000 *g* for 20 min at 4°C. The supernatants were collected and filtered through 0.22-mm polyvinylidene fluoride filters and immediately analyzed. All procedures were carried out at 4°C and were protected from light to avoid potential degradation of the analytes (GSNO and GSH). The LC–ES/MS system consisted of a Waters Allience 2695 HPLC system connected to a Micromass Quattro micro API triple quadrupole mass spectrometer, both obtained from the Waters Corporation. HPLC was carried out using an Atlantis^®^ T3 3 μm 2.1 mm × 100 mm Column obtained from the Waters Corporation. The Micromass Quattro Micro API mass spectrometer was used in positive electrospray ionization mode for detection and quantification of GSNO and GSH (Airaki et al., 2011; Leterrier et al., 2012).

### qRT-PCR Analysis

Roots were ground to a fine powder with a mortar and pestle in liquid nitrogen. Total RNA was extracted using the Tri Reagent solution (Molecular Research Center, Inc., Cincinnati, OH, United States) according to the manufacturer’s instructions. M-MLV reverse transcriptase (Promega, Madison, WI, United States) was used to generate cDNA from 3 μg of DNase-treated root RNA as the template and random hexamers as the primers.

The study of gene expression by qRT-PCR was performed by using a qRT-PCR Bio-Rad CFX connect thermal cycler and the following amplification profile: initial denaturation and polymerase activation (95°C for 3 min), amplification and quantification repeated 40 times (90°C for 10 s, 57°C for 15 s and 72°C for 30 s), and a final melting curve stage of 65 to 95°C with increment of 0.5°C for 5 s, to ensure the absence of primer dimer or non-specific amplification products. PCR reactions were set up in 20 μl of SYBR Green Bio-RAD PCR Master Mix, following the manufacturer’s instructions. Controls containing water instead of cDNA were included to check for contamination in the reaction components. Gene-specific primers (**Table 1**) were designed by using the Primer-BLAST software from the NCBI site. Standard dilution curves were performed for each primer pair to confirm appropriate efficiency of amplification (E = 100 ± 10%). Constitutively expressed *SAND1* and *YLS8* genes, which do not respond to changes in the Fe conditions (Han et al., 2013), were used as reference genes to normalize qRT-PCR results. The relative expression levels were calculated from the threshold cycles (Ct) values and the primer efficiencies by the Pfaffl method (Pfaffl, 2001). Each PCR analysis was conducted on three biological replicates and each PCR reaction repeated twice.

**Table 1:** Primer pairs for *Arabidopsis* genes.

Gene	Sequence 5′–3′
***AtFRO2*** (At1g01580)	**Forward:** TGGTTGCCACATCTGCGTAT **Reverse:** TCGATATGGTGTGGCGACTT
***AtIRT1*** (At4g19690)	**Forward:** TGTCTCTTTTGCAATCTGTCCA **Reverse:** AGGAGCTCCAACACCAATCA
***AtFIT*** (At2g28160)	**Forward:** CCCTGTTTCATAGACGAGAACC **Reverse:** TTCATCTTCTTCACCACCGGC
***AtbHLH38*** (At3g56970)	**Forward:** AAAATGTGTGCATTAGTCCCTT **Reverse:** AGTCTGTGGTACCGTCAATTCAA
***AtGSNOR1*** (At3g56980)	**Forward:** GGTCTCTTTCCTTGTATTCTAG **Reverse:** GCATTCACGACACTCAGCTTG
***AtSAM1*** (At1g02500)	**Forward:** TAATCTCCCATCGAAGCAGCAG **Reverse:** CAACTTTGCTGTCAGGGTCTTG
***AtACO2*** (At1g62380)	**Forward:** AGCAACCCTCTCTCATTCTACA **Reverse:** AGCTTGGACAAGTCTACTACTGG
***AtACS6*** (At4g11280)	**Forward:** ATTGTCTAAAATCGCCTCCGGT **Reverse:** CCACAAAGCTGATTTTCAGCGA
***AtEIN2*** (At5g03280)	**Forward:** ATTCGCACTGATGGGTCTTCTT **Reverse:** CCAAAGATGGCGAACAAATGGT
***AtEIN3*** (At3g20770)	**Forward:** GTCCAGAGCAACCAAACCTCTA **Reverse:** TGTTTCCTGGGAACTGGAGATG
***AtEIL1*** (At2g27050)	**Forward:** CCATCTCTGAAGTTGTGGGGAT **Reverse:** ACCACAATCAAGAACAGAGCCT

### Statistical Analysis

**FIGURE 1:**
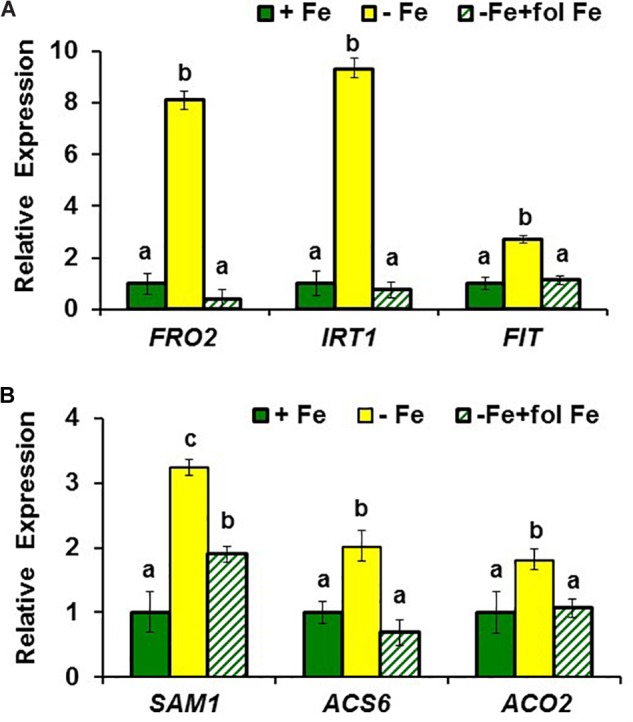
Effect of Fe deficiency and foliar application of Fe on the expression of **(A)** Fe acquisition genes *FRO2, IRT1*, and *FIT*; and **(B)** ethylene synthesis genes *SAM1, ACS6* and *ACO2*, in roots of *Arabidopsis* WT Columbia plants. Plants were grown in nutrient solution with Fe (+Fe). Some of them were transferred to nutrient solution without Fe 3 days before harvest (–Fe). Half of the –Fe plants were sprayed with Fe 24 h before harvest (–Fe+fol Fe). Relative expression was calculated in relation to +Fe. Data represent the mean ± SE of three independent biological replicates. Within each gene, bars with different letters indicate significant differences (*P* < 0.05).

**FIGURE 2:**
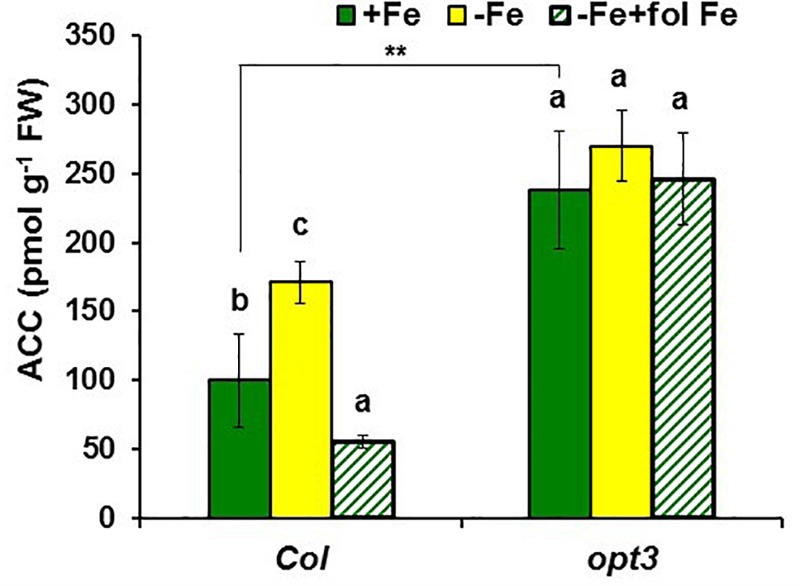
Effect of Fe deficiency and foliar application of Fe on ACC content in roots of *Arabidopsis* WT Columbia and *opt3-2* mutant plants. Treatments as in **Figure 1**. Data represent the mean ± SE of six replicates. Within each genotype, bars with different letters indicate significant differences (*P* < 0.05). Significant difference between the +Fe treatment from *opt3-2* and Col is also indicated: ^∗∗^*P* < 0.05.

**FIGURE 3:**
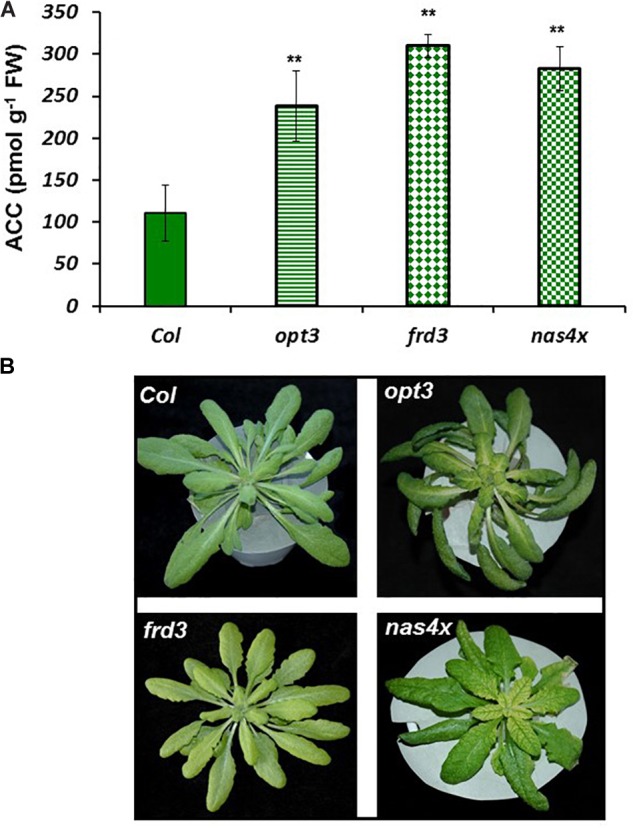
ACC concentration in Fe-sufficient roots **(A)** and shoot appearance **(B)** of *Arabidopsis* WT Columbia and *opt3-2, frd3-3*, and *nas4x-1* mutant plants. Note the chlorosis in the *frd3-3* and *nas4x-1* mutants. Plants were grown in nutrient solution with Fe. Data represent the mean ± SE of six replicates. Significant differences from Col are indicated: ^∗∗^*P* < 0.05.

All experiments were repeated at least twice and representative results are presented. The values of qRT-PCR represent the mean ± SE of three independent biological replicates. The values of other determinations (ACC, GSNO, GSH, ferric reductase activity, GSNOR activity) represent the mean ± SE of six replicates. Within each gene or genotype, different letters indicate significant differences (*P* < 0.05) among treatments using one-way analysis of variance (ANOVA) followed by a Duncan’s multiple range test (i.e., **Figures 1, 2, 6**). Dunnett’s test was also used when one or several mutants were compared with the WT for the +Fe treatment (i.e., **Figures 2, 3**) and when different treatments were compared with a control (i.e., **Figures 3, 9**). In this latter case, ^∗∗^ indicate significant differences (*P* < 0.05).

## Results

### Effect of LODIS on Ethylene Synthesis in Roots

To observe whether LODIS affects ethylene synthesis in roots, we determined ACC (ethylene precursor) concentration and the expression of several genes implicated in ethylene synthesis (*SAM1, ACS6*, and *ACO2*; García et al., 2010) in roots of *Arabidopsis* WT Columbia plants grown under different Fe treatments that could affect LODIS accumulation: with Fe (LODIS sufficient), without Fe (LODIS deficient), and without Fe but with Fe sprayed on leaves (LODIS sufficient). The expression of all the ethylene synthesis genes studied and ACC production greatly increased (sometimes, more than 2-fold) in Fe-deficient Columbia roots, in parallel to the expression of the key Fe acquisition genes *FRO2, IRT1*, and *FIT* (**Figures 1, 2**). On the other hand, the foliar application of Fe to Fe-deficient Columbia plants greatly repressed the expression of Fe acquisition (**Figure 1A**) and ethylene synthesis genes (**Figure 1B**), and ACC production (**Figure 2**), which suggests that LODIS inhibits ethylene synthesis. This inhibitory effect of LODIS was further supported by the fact that the foliar application of Fe did not significantly inhibit ACC production in the *Arabidopsis* LODIS-deficient mutant *opt3-2* (**Figure 2**; García et al., 2013; Zhai et al., 2014). This mutant also showed higher ACC concentration in Fe-sufficient roots compared to the WT Columbia (**Figure 2**), which suggests that total Fe in roots does not control ethylene synthesis. Similar higher ACC levels were found in Fe-sufficient roots of other *Arabidopsis* mutants that behave like LODIS-deficient (*frd3-3* and *nas4x-1*) and that present constitutive activation of Fe acquisition genes under Fe-sufficiency (**Figure 3A**; García et al., 2013; see section “Materials and Methods”).

It should be noted that, although the *frd3-3* and *opt3-2* mutants usually accumulate more metals than the WT cultivar (Rogers and Guerinot, 2002; Stacey et al., 2008), neither of them showed any symptom of metal toxicity in our experimental conditions (**Figure 3B**). The *frd3-3* and *nas4x-1* mutants showed chlorosis, as expected (**Figure 3B**; see section “Materials and Methods”).

**FIGURE 4:**
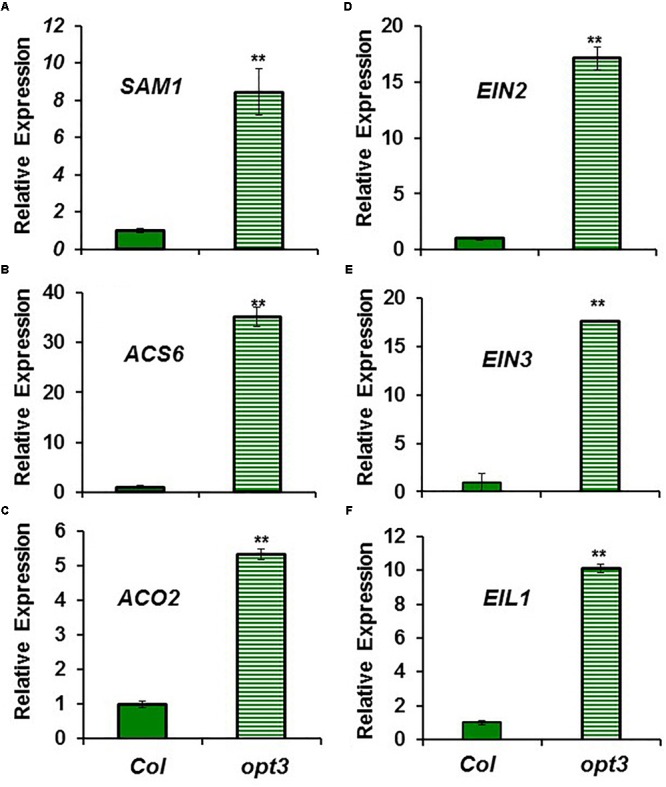
Expression of ethylene synthesis genes **(A)** *SAM1*, **(B)** *ACS6* and **(C)** *ACO2*, and ethylene signaling genes **(D)** *EIN2*, **(E)** *EIN3* and **(F)** *EIL1*, in Fe-sufficient roots of *Arabidopsis* WT Columbia and *opt3-2* mutant plants. Plants were grown in nutrient solution with Fe. Relative expression was calculated in relation to Col. Data represent the mean ± SE of three independent biological replicates. Significant differences between *opt3-2* and Col are indicated: ^∗∗^*P* < 0.05.

The higher ACC content in Fe-sufficient *opt3-2* roots than in Columbia roots (**Figure 2**) was correlated with higher expression of the ethylene synthesis genes *SAM1, ACS6* and *ACO2*, and of the ethylene signaling genes *EIN2, EIN3* and *EIL1*, in the mutant (from 5- to 30-fold; **Figure 4**). Collectively, these results suggest that ethylene (synthesis and signaling) drastically increases under low LODIS accumulation in roots (i.e., Fe deficiency in WT or Fe sufficiency in the LODIS-deficient mutant *opt3-2*).

### Effect of LODIS on GSNOR Expression and Activity in Roots

**FIGURE 5:**
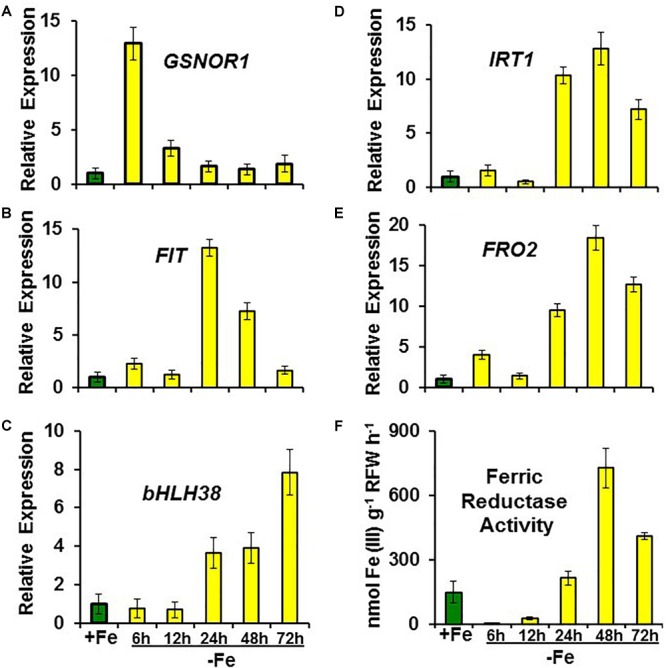
Time course experiment of the effect of Fe deficiency on the expression of **(A)** *GSNOR1*, Fe acquisition genes **(B)** *FIT*, **(C)** *bHLH38*, **(D)** *IRT1* and **(E)** *FRO*2), and on **(F)** ferric reductase activity, in roots of *Arabidopsis* WT Columbia plants. Plants were grown in nutrient solution with Fe (+Fe). Some of them were transferred to nutrient solution without Fe (–Fe) 6, 12, 24, 48, or 72 h before determination of ferric reductase activity or harvest. For genes, data represent the mean ± SE of three independent biological replicates. Relative expression was calculated in relation to +Fe. For ferric reductase activity, data represent the mean ± SE of six replicates.

**FIGURE 6:**
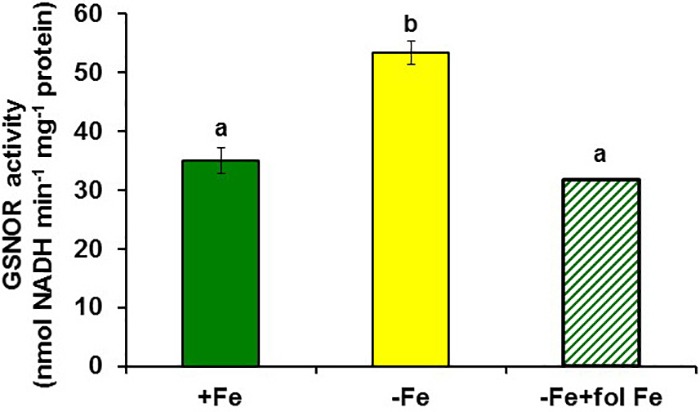
Effect of Fe deficiency and foliar application of Fe on GSNOR activity in roots of *Arabidopsis* WT Columbia plants. Plants were grown in nutrient solution with Fe (+Fe) and some of them were transferred to nutrient solution without Fe 2 days before harvest (–Fe). Half of the –Fe plants were sprayed with Fe 24 h before harvest (–Fe+fol Fe). Data represent the mean ± SE of six replicates. Bars with different letters indicate significant differences (*P* < 0.05).

**FIGURE 7:**
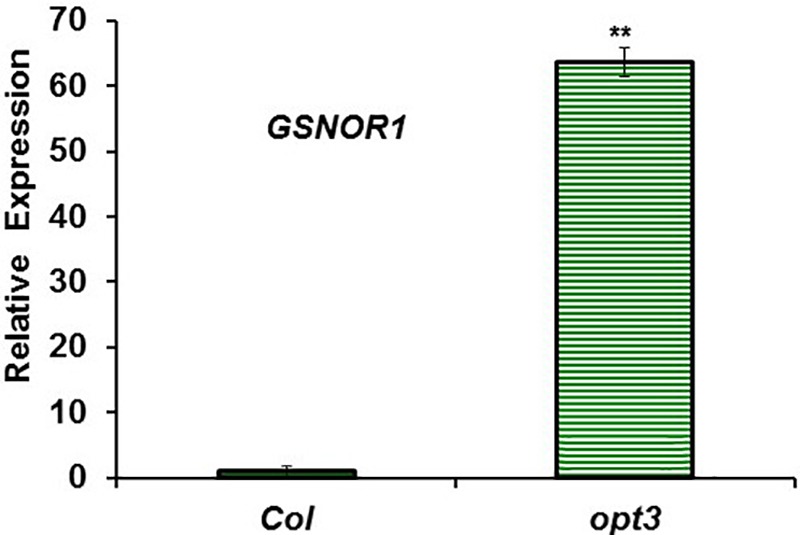
*GSNOR1* expression in roots of Fe-sufficient *Arabidopsis* WT Columbia and *opt3-2* mutant plants. Plants were grown in nutrient solution with Fe. Relative expression was calculated in relation to Col. Data represent the mean ± SE of three independent biological replicates. Significant difference between *opt3-2* and Col is indicated: ^∗∗^*P* < 0.05.

**FIGURE 8:**
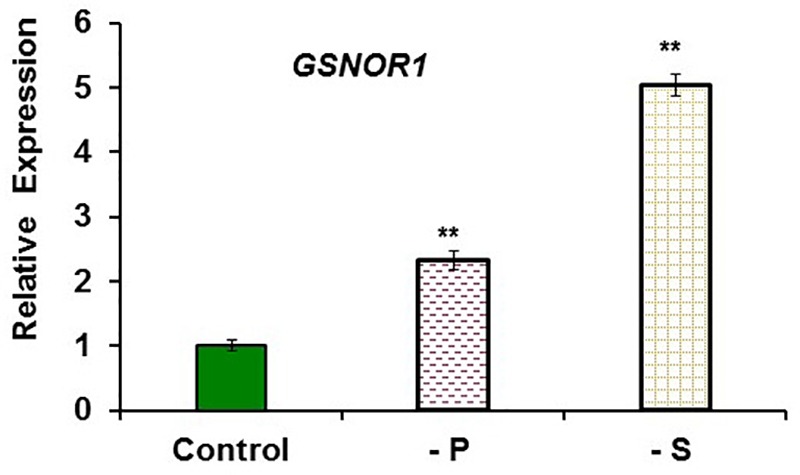
Effect of P or S deficiency on *GSNOR1* expression in roots of *Arabidopsis* WT Columbia plants. Plants were grown in complete nutrient solution (Control). Some of them were transferred to nutrient solution without P (–P) or without S (–S) during the last 2 days. Relative expression was calculated in relation to Control. Data represent the mean ± SE of three independent biological replicates. Significant differences from the Control treatment are indicated: ^∗∗^*P* < 0.05.

**FIGURE 9:**
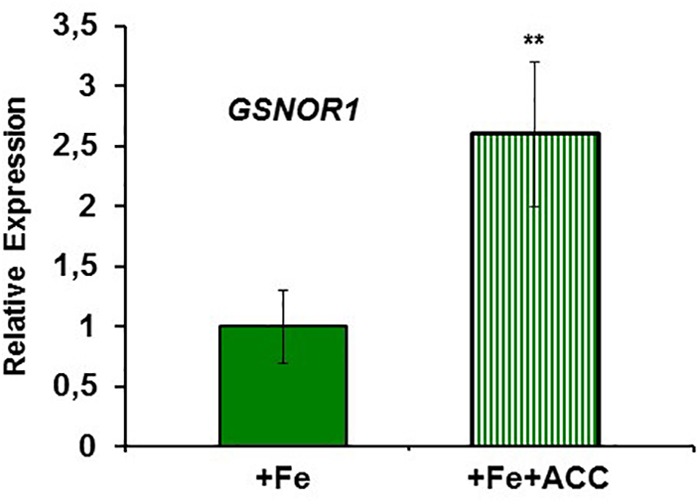
Effect of ACC on *GSNOR1* expression in roots of *Arabidopsis* WT Columbia plants. Plants were grown in nutrient solution with 40 μM Fe (+Fe) and half of them treated with 1 μ M ACC, final concentration (+Fe+ACC), 24 h before harvest. Relative expression was calculated in relation to +Fe. Data represent the mean ± SE of three independent biological replicates. Significant differences from the +Fe treatment are indicated: ^∗∗^*P* < 0.05.

To study the possible relationship of GSNOR with Fe deficiency, we analyzed GSNOR expression and activity under different Fe treatments and genetic backgrounds that could influence LODIS levels. The results showed that *GSNOR1* expression increased (more than 10-fold) under Fe deficiency in roots of the WT cultivar Columbia (**Figure 5A**). This up-regulation of *GSNOR1* occurred very quickly, after few hours of Fe depletion, and slightly before the up-regulation of the main regulator of the Fe deficiency responses, *FIT* (**Figure 5B**), other Fe acquisition genes (*bHLH38, IRT1, FRO2*; **Figures 5C–E**), and the enhancement of ferric reductase activity (**Figure 5F**). In agreement with the *GSNOR1* up-regulation, the GSNOR activity significantly increased (approximately 50%) in Fe-deficient Columbia roots (**Figure 6**). This activity was restored to normal level upon foliar application of Fe (**Figure 6**), which suggests that it could be inhibited by the accumulation of LODIS in roots. To further confirm this possibility, we compared *GSNOR1* expression in Fe-sufficient WT and *opt3-2* roots. As shown in **Figure 7**, *GSNOR1* expression was much higher (more than 50-fold) in Fe-sufficient *opt3-2* roots (LODIS-deficient) than in Fe-sufficient Columbia roots, which suggests that its expression depends on LODIS and not on total root Fe content. The up-regulation of *GSNOR1* expression also occurred under other nutrient deficiencies, such as P deficiency and S deficiency (**Figure 8**). Furthermore, *GSNOR1* expression was up-regulated by ACC treatment in Fe-sufficient Columbia roots (**Figure 9**).

**FIGURE 10:**
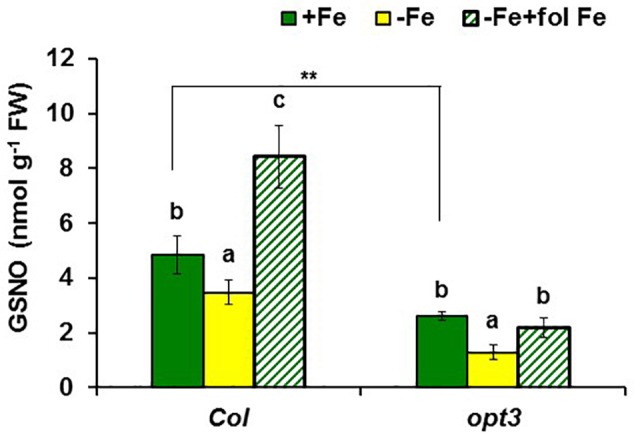
Effect of Fe deficiency and foliar application of Fe on GSNO content in roots of *Arabidopsis* WT Columbia and *opt3-2* mutant plants. Plants were grown in nutrient solution with Fe (+Fe). Some of them were transferred to nutrient solution without Fe 2 days before harvest (–Fe). Half of the –Fe plants were sprayed with Fe 24 h before harvest (–Fe+fol Fe). Data represent the mean ± SE of six replicates. Within each genotype, bars with different letters indicate significant differences (*P* < 0.05). Significant difference between the +Fe treatment from *opt3-2* and Col is also indicated: ^∗∗^*P* < 0.05.

**FIGURE 11:**
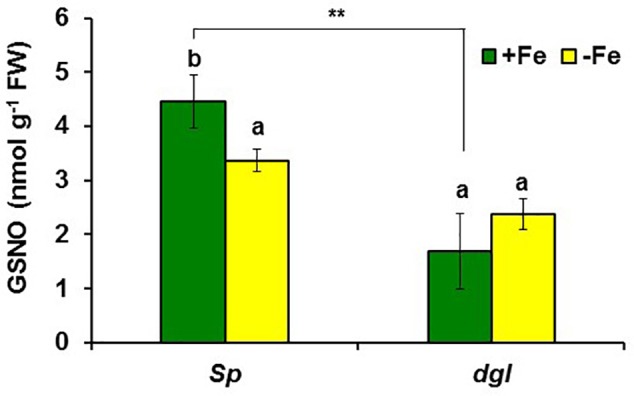
Effect of Fe deficiency on GSNO content in roots of pea WT Sparkle and *dgl* mutant plants. Plants were grown in nutrient solution with Fe (+Fe) and half of them were transferred to nutrient solution without Fe 2 days before harvest (–Fe). Data represent the mean ± SE of six replicates. Within each genotype, bars with different letters indicate significant differences (*P* < 0.05). Significant difference between the +Fe treatment from *dgl* and Sparkle is also indicated: ^∗∗^*P* < 0.05.

In addition to GSNOR, we also determined GSNO concentration in roots of *Arabidopsis* WT Columbia and *opt3-2* plants subjected to different Fe treatments. The results showed a decrease of approximately 30% of GSNO content in Fe-deficient WT roots and, by contrast, a great increase upon foliar application of Fe (**Figure 10**). GSNO also decreased in Fe-deficient *opt3-2* roots but was less affected by the foliar application of Fe (**Figure 10**). The lower GSNO content in Fe-deficient roots could be explained by the higher GSNOR expression and activity in these roots (**Figures 5, 6**). In supporting this view, Fe-sufficient *opt3-2* roots, which had higher *GSNOR* expression than Fe-sufficient WT roots (**Figure 7**), had lower GSNO content (approximately 50%; **Figure 10**). Similar results were also found with the pea LODIS-deficient mutant *dgl* and its WT cultivar Sparkle. As shown in **Figure 11**, GSNO content also decreased in Fe-deficient Sparkle roots (approximately 25%) and, similarly to *opt3-2*, GSNO content was lower in Fe-sufficient *dgl* roots (approximately 40%) than in Fe-sufficient Sparkle roots.

Collectively, all these results suggest that GSNOR (expression and activity) increases, and consequently GSNO decreases, under low LODIS accumulation in roots (i.e., Fe deficiency in WT or Fe sufficiency in LODIS-deficient mutants).

### Effect of Fe Deficiency on GSH Content in Roots and Leaves of WT and *opt3-2* Mutant Plants

In our experimental conditions, GSH greatly increased in Fe-deficient WT roots but hardly in Fe-deficient *opt3-2* roots (**Table 2**). In leaves, the most noticeable result was the higher GSH content in the *opt3-2* mutant in relation to Columbia under Fe sufficiency (**Table 2**).

**Table 2:** Effect of Fe deficiency on GSH (glutathione) concentration in roots and leaves of *Arabidopsis* WT Columbia and *opt3-2* mutant plants.

Compound	Genotype	Roots		Leaves	
		**+Fe**	–**Fe**	**+Fe**	–**Fe**
GSH (nmol⋅g^-1^FW)	Col	53 ± 4	144 ± 1^∗∗^	146 ± 10	137 ± 8
	*opt3-2*	43 ± 9	60 ± 15	286 ± 12	138 ± 3^∗∗^

*Plants were grown in nutrient solution with Fe (+Fe) and half of them transferred to nutrient solution without Fe (-Fe) during the last 2 days. Data represent the mean ± SE of six replicates. Significant differences from the +Fe treatment are indicated: ^∗∗^*P* < 0.01.*

## Discussion

Ethylene increases in Fe-deficient roots, where it activates the expression of key Fe acquisition genes (reviewed in Lucena et al., 2015; Li and Lan, 2017; Romera et al., 2017). A signal related to phloem Fe, LODIS, blocks this activating effect (García et al., 2013). However, it is not known whether LODIS could affect ethylene synthesis in roots. Results presented in this work (**Figures 1, 2, 3A, 4**) support that LODIS does affect ethylene synthesis. The foliar application of Fe greatly decreased the expression of Fe acquisition and ethylene synthesis genes (**Figure 1**), and ACC production (**Figure 2**), in Fe-deficient WT Columbia roots. However, in Fe-deficient roots of the LODIS-deficient mutant *opt3-2* (García et al., 2013; Mendoza-Cózatl et al., 2014; Zhai et al., 2014), foliar application of Fe had almost no effect on ACC content of roots (**Figure 2**). Moreover, the expression of several ethylene synthesis and signaling genes was much higher in Fe-sufficient *opt3-2* roots than in Fe-sufficient Columbia roots (**Figure 4**). In the same way, Fe-sufficient roots of *opt3-2* and other *Arabidopsis* mutants that behave as LODIS-deficient, such as *frd3-3* and *nas4x-1* (see section “Materials and Methods”), had greater ACC content than Fe-sufficient Columbia roots (**Figures 2, 3A**). All these results suggest that ethylene synthesis (and signaling) in roots does not depend on total root Fe content (these mutants accumulate high levels of Fe inside roots under Fe sufficiency; García et al., 2013 and references therein) but on LODIS levels, that depend on the OPT3 transporter.

Since heavy metals can enhance ethylene production and the expression of ethylene signaling genes (Keunen et al., 2016), it can be argued that the higher ACC content and the higher expression of ethylene-related genes in Fe-sufficient *opt3-2* roots (and in Fe-sufficient *frd3-3* and *nas4x-1* roots) could be caused by the high accumulation of metals described in some of these mutants. Against this argument, we have to say that, in our experiments, plants were grown with low levels of metals and they did not present leaf toxicity symptoms (**Figure 3B**). Moreover, after the work of Vert et al. (2002) it is known that the accumulation of metals in mutants that present constitutive activation of *IRT1* expression, such as *opt3-2* (Stacey et al., 2008) and *frd3* (Rogers and Guerinot, 2002), is mainly due to the broad substrate range of this transporter. IRT1 can transport several divalent metals, besides Fe^2+^, such as Mn^2+^, Zn^2+^, or Cd^2+^ (Korshunova et al., 1999; Lucena et al., 2006). Since *IRT1* expression is activated by ethylene (García et al., 2010; Lingam et al., 2011; Blum et al., 2014; Marín-de la Rosa et al., 2014), the most probable sequence is: (1) enhanced ethylene production → (2) activation of *IRT1* expression → (3) accumulation of metals, and not: (1) accumulation of metals → (2) enhanced ethylene production → (3) activation of *IRT1* expression. No accumulation of metals would occur without IRT1 activity. In supporting this view, it should be noted that the inhibition of ethylene synthesis with cobalt, a potent ethylene inhibitor, drastically inhibited *IRT1* expression and the accumulation of metals in tomato plants (Lucena et al., 2006) and in pea *brz* mutant plants (also named E107, has constitutive activation of Fe responses; Romera et al., 1996, 2015). Taken together, all these results suggest that ethylene is necessary for *IRT1* up-regulation and, consequently, for the acquisition and accumulation of metals. This does not discard that, if plants accumulate high levels of metals, they could cause a further increase of ethylene production, but as a later side effect.

Similarly to ethylene, GSH (**Table 2**) and NO also increase in Fe-deficient WT roots, where they have been implicated in the activation of Fe deficiency responses (Zaharieva and Abadía, 2003; Zaharieva et al., 2004; Graziano and Lamattina, 2007; Bacaicoa et al., 2009; Chen et al., 2010; García et al., 2011; Koen et al., 2012; Shanmugam et al., 2015; Kailasam et al., 2018). However, results in this work show that GSNO content in WT roots, in contrast to its precursors GSH and NO, decreases upon Fe deficiency (**Figures 10, 11**), which agrees with previous results (Shanmugam et al., 2015; Kailasam et al., 2018). Since GSNOR reduces GSNO to GSSG and other byproducts, like hydroxylamine and H_3_N (Leterrier et al., 2011; Corpas et al., 2013; Kubienová et al., 2014), we wanted to know whether GSNOR increases in Fe deficient roots. In fact, *GSNOR1* expression and activity in roots increased early after Fe deficiency and slightly before the up-regulation of Fe acquisition genes (**Figures 5, 6**). This suggests that GSNOR could be responsible for the lower GSNO contents found in Fe-deficient WT roots (**Figures 10, 11**; Shanmugam et al., 2015; Kailasam et al., 2018). *GSNOR1* is highly responsive to the Fe status of the plant but, as for ethylene (see above), the results indicate that its up-regulation does not depend on the total root Fe content but on LODIS levels. First, the foliar application of Fe greatly increased GSNO content in Fe-deficient Columbia roots (probably by decreasing GSNOR activity, as seen in **Figure 6**) but hardly in Fe-deficient roots of the LODIS-deficient mutant *opt3-2* (**Figure 10**), which is impaired in the movement of Fe from shoots to roots (García et al., 2013; Mendoza-Cózatl et al., 2014; Zhai et al., 2014). Second, Fe-sufficient *opt3-2* roots presented much higher *GSNOR1* expression (and less GSNO content) than Fe-sufficient Columbia roots (**Figures 7, 10**). Similarly, a lower GSNO content was found in Fe-sufficient roots of the pea LODIS-deficient mutant *dgl* than in Fe-sufficient roots of its WT Sparkle (**Figure 11**). All these results clearly suggest that GSNOR expression (and consequently GSNO content) does not depend on total root Fe content but on LODIS levels.

The results described above clearly show that the higher NO accumulation found in Fe-deficient WT roots (Graziano and Lamattina, 2007; Chen et al., 2010; García et al., 2011) is associated with lower GSNO contents (**Figures 10, 11**). Similar results have been found by others in roots of *Arabidopsis* plants subjected to Fe deficiency (Shanmugam et al., 2015; Kailasam et al., 2018), to arsenic stress (Leterrier et al., 2012) or to Cd stress (Liu et al., 2018). In the two latter cases, the lower GSNO levels have been associated with a higher GSNOR activity (Leterrier et al., 2012; Liu et al., 2018), which also occurs under other abiotic stress conditions (Corpas et al., 2008; Airaki et al., 2012; Kubienová et al., 2014; Cheng et al., 2015). In relation to Cd stress, it is important to mention that Cd can cause Fe deficiency (Alcántara et al., 1994 and references therein) and induce Fe deficiency responses (Yoshihara et al., 2006). In our work, besides Fe deficiency, *GSNOR1* expression also increased under P or S deficiency (**Figure 8**), which indicates that GSNOR could be implicated in the responses to different nutritional deficiencies. The higher NO accumulation associated with a lower GSNO content in Fe-deficient roots suggests that NO and GSNO do not have the same exact roles, probably because they have different lifetime and location. NO lifetime is relatively short (less than 10 s) while GSNO is a relatively stable store for NO and might constitute a vehicle for its long-distance transport (Corpas et al., 2011; Malik et al., 2011), mediating a signaling function throughout a process of *S*-transnitrosation between GSNO and proteins (Corpas et al., 2013). In relation to their location, both NO and GSNO can present different cellular and intracellular location (Malik et al., 2011; Shi et al., 2015). NO production is associated with phloem cells (Corpas et al., 2004; Gaupels et al., 2008) and, since the GSNOR protein is also located in the phloem (Rustérucci et al., 2007), it is possible that GSNO could be a vehicle to transmit the NO-signal out of the phloem. In agreement with the distinct role of NO and GSNO, Yun et al. (2016) have found that both of them act differently in the responses associated with plant immunity. In relation to Fe deficiency responses in dicot plants, Kailasam et al. (2018) have recently shown, by using chemical screening, that GSNO probably affects the regulation of Fe acquisition genes through FIT while NO could affect them through other TFs, such as bHLH38 and bHLH39. It should be noted that the SKB1 protein, implicated in *bHLH38* and *bHLH39* expression (Fan et al., 2014), can be nitrosylated (Hu et al., 2015). In this way, it is possible that GSNO, through the nitrosylation/de-nitrosylation of SKB1, could affect the expression of these Ib bHLH TFs (Fan et al., 2014; Lucena et al., 2015). In supporting this view, *bHLH38, bHLH39*, and *bHLH100* are up-regulated in leaves of the *Arabidopsis gsnor1-3* mutant, which has altered GSNO content (Xu et al., 2013).

**FIGURE 12:**
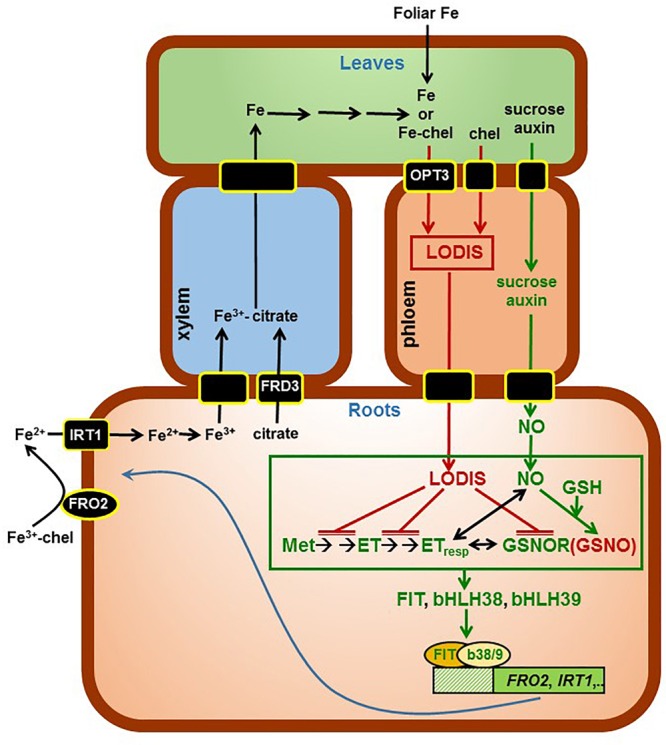
Working Model to explain the role of LODIS on the regulation of Fe acquisition genes. Once inside roots, Fe is translocated to leaves through the xylem, bound to citrate (provided by the FRD3 transporter). In shoots, some Fe (either as free ions or in chelated form) can enter the phloem through the OPT3 transporter, and moves back to roots bound to a chelating agent (forming LODIS). In roots, LODIS could negatively affect ethylene synthesis and signaling, and GSNOR expression and/or activity, which can lead to enhanced GSNO. Besides LODIS, which would act as a repressor of Fe responses, some other shoot signals, like sucrose and auxin, would act as activators of Fe responses through NO (Lin et al., 2016). The possible relationship of ethylene, NO, GSH, GSNOR, and GSNO is depicted in more detail in **Figure 13**. In green are components whose expression, activity and/or content is known to increase under Fe deficiency while in red are components whose expression, activity, and/or content is known to increase under Fe sufficiency. chel, chelating agent; GSH, glutathione; GSNO, *S*-nitrosogluthatione; GSNOR, GSNOR reductase; ET, ethylene; ETresp, ethylene response; Met, methionine (→: promotion; $\begin{array}{c} \bar{T} \end{array}$: inhibition).

Results in this work, along with previous results (García et al., 2013), suggest that LODIS can inhibit both ethylene synthesis and signaling, and GSNOR expression and activity in roots (see Working Model in **Figure 12**). The inhibition of GSNOR would increase GSNO content in roots, which agrees with the higher GSNO contents found in Fe-sufficient WT roots or in roots of Fe-deficient WT plants treated with foliar Fe (**Figures 10, 11**; Shanmugam et al., 2015; Kailasam et al., 2018). After all these results, several questions arise: what is the nature of LODIS?; is there any relationship between GSNO(NO), GSH and ethylene?; is there any relationship between GSNO(NO)/ethylene and the putative Fe sensor BTS? In relation to the nature of LODIS, it is unknown at present. Although Zhai et al. (2014) found that OPT3 can transport Fe^2+^ ions when expressed in *Xenopus* oocytes, the substrate of OPT3 remains controversial because the Fe transport function of OPT3 could not be confirmed in other experiments (Mendoza-Cózatl et al., 2014). Even if Fe enters the phloem through OPT3 as free ions, it would be difficult for Fe to move in this way, and probably the Fe ions would be chelated (**Figure 12**; see section “Introduction”). OPT3 does not mediate GSH transport in *S. cerevisiae* (Zhai et al., 2014), as other OPT transporters did (Zhang et al., 2016 and references therein). However, the higher leaf GSH content found in the *opt3-2* mutant in relation to Columbia, under Fe sufficiency (**Table 2**) or under Cd treatment (Akmakjian, 2011), supports the idea of LODIS being a compound related, directly or indirectly, to GSH, such as GS-Fe-NO or NICs (Ramírez et al., 2011; Rahmanto et al., 2012; Darbani et al., 2013; Buet and Simontacchi, 2015). Nonetheless, this idea deserves further research.

**FIGURE 13:**
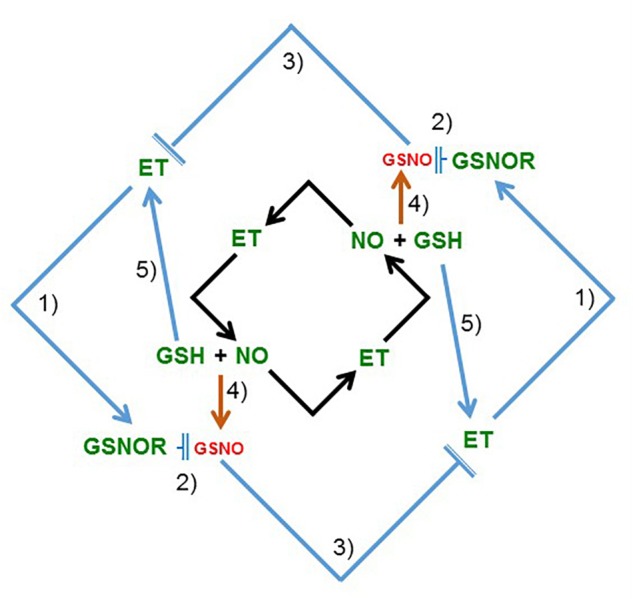
Model to explain the relationship between ethylene and NO, and between ethylene and GSNOR (GSNO), in roots. In previous works, and also in this work (see **Figure 2** and **Table 2**), it has been shown that ethylene, NO, and GSH increase in Fe-deficient roots of dicot plants (see section “Introduction”). Moreover, it has been shown that ethylene can influence NO accumulation in roots and vice versa (black arrows; García et al., 2011; Liu et al., 2017). In this work, it has been found that ethylene and GSNOR increase under low LODIS accumulation in roots (see **Figure 12**). After that, a new model, complementary of the previous one, is proposed (blue arrows) suggesting mutual possible interactions among ethylene, GSNOR, GSH and NO: (1) ethylene can upregulate GSNOR (see **Figure 9**); (2) GSNOR can decrease GSNO (Corpas et al., 2013); (3) Low GSNO can unblock ethylene synthesis since nitrosylation of SAM synthetase by GSNO can inhibit ethylene synthesis (Lindermayr et al., 2006; Freschi, 2013; see section “Discussion”); (4) GSNO derives from NO and GSH (Corpas et al., 2013); (5) GSH can enhance ethylene production (Datta et al., 2015). In green are components whose expression, activity and/or content is known to increase under Fe deficiency while in red are components whose content is known to decrease under Fe deficiency. GSH, glutathione; GSNO, *S*-nitrosoglutathione; GSNOR, GSNO reductase; ET, ethylene; NO, nitric oxide (→: promotion; $\begin{array}{c} \bar{T} \end{array}$: inhibition).

In relation to the possible interactions between GSNO(NO), GSH and ethylene, it should be said that, besides the mutual and positive influence between NO and ethylene already described in previous works (**Figure 13**; García et al., 2011; Romera et al., 2011; Liu et al., 2017), GSNO(NO), GSH and ethylene could also interact at other levels. GSH can enhance ethylene production by affecting some ethylene synthesis enzymes (Datta et al., 2015). On the other hand, the relationship between GSNO and ethylene can be feasible because both GSNOR (Rustérucci et al., 2007; see above) and several ethylene synthesis enzymes, like 5-methilthioribose kinase (induced in Fe-deficient roots; García et al., 2010; Romera et al., 2017 and references therein), are located in the phloem (Pommerrenig et al., 2011). Moreover, enzymes implicated in ethylene synthesis, such as SAM synthetases (also named methionine adenosyltransferases; Sauter et al., 2013), can be inhibited by *S*-nitrosylation (Lindermayr et al., 2006; Freschi, 2013). Since GSNO has been implicated in the reversible *S*-nitrosylation of proteins (Malik et al., 2011; Begara-Morales et al., 2015; Zaffagnini et al., 2016), it is possible that higher GSNO levels (such as those found in Fe-sufficient WT roots: **Figures 10, 11**) could contribute to *S*-nitrosylation of ethylene synthesis enzymes and, consequently, to the inhibition of ethylene synthesis (**Figure 13**). By contrast, lower GSNO levels (such as those found in Fe-deficient WT roots or in Fe-sufficient *opt3-2* and *dgl* roots: **Figures 10, 11**) could contribute to de-nitrosylation of ethylene synthesis enzymes and, consequently, to promotion of ethylene synthesis (**Figures 1B, 2**). In supporting this view, silencing *GSNOR* in *Nicotiana attenuata*, which leads to higher GSNO content, decreased the herbivore-induced accumulation of ethylene (Wünsche et al., 2011). Reciprocally to the possible influence of GSNO(NO) on ethylene synthesis, ethylene (ACC) could decrease GSNO content by activating *GSNOR1* expression (**Figures 9, 13**). *GSNOR1* expression is also influenced by other hormones, such as salicylic acid which activates it or jasmonic acid which down-regulates it (Díaz et al., 2003). Very recently, it has been found that ethylene (ACC) can increase NO content by activating enzymes involved in its synthesis, such as nitrate reductase and nitric oxide synthase-like (Liu et al., 2017). This would imply that ethylene could simultaneously increase NO accumulation and decrease GSNO content (**Figure 13**).

Finally, in the relationship between GSNO(NO)/ethylene and the putative Fe sensor BTS, some results suggest that the BTS protein could act upstream of ethylene. The *SAM1* gene, encoding a SAM synthetase (involved in ethylene synthesis; Sauter et al., 2013), is up-regulated in the *bts-3* mutant (Hindt et al., 2017). In the same way, several genes whose expression is activated by ethylene, such as *FIT, bHLH38, bHLH39, MYB72, BGLU42, FRO2, IRT1, FRD3*, and *NAS2* (García et al., 2010), are up-regulated in *bts-3* roots under Fe-sufficient conditions (Hindt et al., 2017). In rice, roots from HRZ-knockdown plants have higher jasmonate content than the WT ones when grown under Fe sufficiency (Kobayashi et al., 2016). All these results suggest that BTS/HRZs may act as sensors of LODIS, as suggested by Kobayashi and Nishizawa (2014), and depending on its binding state could then affect the synthesis of hormones, like ethylene or jasmonate.

## Conclusion

The results presented in this work, along with already published results, suggest that shoots, through LODIS, play an important role in the regulation of Fe acquisition genes in roots, which is depicted in the Working Model of **Figure 12**. According to this model, under Fe sufficiency, sufficient Fe goes to leaves and enters the phloem to form LODIS, which travels to roots and represses ethylene synthesis and signaling, and *GSNOR* expression and activity, which leads to increased GSNO content. GSNO(NO), GSH, and ethylene can interact in different ways (see above paragraph and **Figure 13**). Moreover, a high GSNO content can compromise auxin signaling and transport (Shi et al., 2015), which is important because auxin has also been involved in the regulation of Fe responses, where is intimately interrelated with ethylene and NO (Chen et al., 2010; Romera et al., 2011, 2017). Under Fe deficiency, the lack of LODIS derepresses ethylene synthesis and signaling, and *GSNOR* expression and activity, which leads to decreased GSNO content. All this would cause the up-regulation of Fe acquisition genes.

This working model could open the way to better understand the role of shoots and roots in the regulation of Fe deficiency responses. According to this model, LODIS would have a double role in the regulation of Fe acquisition genes, by controlling both ethylene synthesis and signaling (**Figure 12**). This double role of LODIS would explain why the ethylene overproducer mutant *eto* and the treatments with ethylene did not activate the expression of Fe acquisition genes when plants are grown with high levels of Fe (Lucena et al., 2015): despite high ethylene levels, LODIS would block ethylene action. All the results discussed in this work imply that the relationship of GSH, NO and GSNO with ethylene, with GSNOR, with LODIS and with the expression of Fe acquisition genes is complex and deserves further research.

## Author’s Note

A very recent work by Grillet et al. (2018) shows that some peptides (IRON MAN or IMA peptides) associated with the phloem, and preferentially expressed in leaves, could be implicated in the regulation of Fe responses in roots. The upregulation of Fe responses in lines overexpressing genes encoding these peptides and their ability to bind Fe^2+^ suggest that these peptides could act either as activators (unbound IMA peptides) or repressors (IMA peptides bound to Fe) of the responses. Perhaps LODIS is related to the ratio between IMA peptides bound to Fe and unbound IMA peptides.

## Author Contributions

FR and MG designed the experiments after discussions with PB, RP-V, and EA. MG and CL conducted the laboratory work. FC and MG determined GSNOR, GSNO, and GSH. ÁZ, EB, and JG-M determined ACC. FR, MG, RP-V, and EA wrote the manuscript that was improved by the other authors.

## Conflict of Interest Statement

The authors declare that the research was conducted in the absence of any commercial or financial relationships that could be construed as a potential conflict of interest.
